# Functional Outcomes of Displaced Midshaft Clavicular Fractures Treated with Precontoured Locked Plates: A Prospective Study

**DOI:** 10.12688/f1000research.162891.2

**Published:** 2025-06-25

**Authors:** Abdullah Ali Al-Moaish, Jamal Abdulraheem Algabarty, Anwar Mughallas, Ali Mustafa Alhamzi, Mosleh Soliaman, Mohammed Hutaif, Mohammed Abdulmoghni, Abdukareem Hussain Almahdi, Haitham Mohammed Jowah

**Affiliations:** 1 Department of Orthopedic Surgery, Al-Thawra Modern General Hospital, Sana'a, Yemen; 2 Department of Surgery, Sana'a University, Sana'a, Yemen; 3 Department of Orthopedic Surgery, Kuwait University Hospital, Sana'a, Yemen

**Keywords:** clavicle fracture, precontoured locked plate, functional outcomes, surgical management, complications, patient satisfaction, midshaft fracture, ORIF

## Abstract

**Background:**

This study assessed the functional outcomes and complications of open reduction and internal fixation (ORIF) using pre-contoured superior clavicle locking plates for displaced midshaft clavicular fractures.

**Methods:**

In a prospective two-center study at Al-Thawra Modern General Hospital and Kuwait University Hospital, Sana’a, Yemen, from January 2018 to September 2024, 65 patients (≥18 years) with closed, displaced midshaft clavicular fractures (displacement >2 cm, shortening >2 cm, comminution, or skin tenting) underwent ORIF. Functional outcomes were evaluated six months postoperatively using the University of California, Los Angeles (UCLA) shoulder rating score. Data were analyzed using SPSS version 26.

**Results:**

The mean patient age was 32.09 years (83.1% male, n=54). Road traffic accidents were the primary mechanism of injury (66.2%, n=43). At 6 months, the mean UCLA score was 32.46 ± 2.54, with 98.5% (n=64) achieving good or excellent outcomes (UCLA score ≥27) and 1.5% (n=1) fair/poor. Complications included hardware irritation (1.5%, n=1), hardware failure (3.1%, n=2), and superficial infections (1.5%, n=1). All patients (100%) reported satisfaction with their outcomes. The UCLA scores varied significantly according to injury mechanism, side, and age, with older patients showing lower scores.

**Conclusion:**

ORIF with pre-contoured locked plates yielded promising functional outcomes, high patient satisfaction, and low complication rates. However, the observational design, lack of a control group, and 6-month follow-up limit broader conclusions. Larger controlled studies are needed to validate these findings and guide the optimal management of displaced midshaft clavicular fractures.

## Introduction

Clavicular fractures, comprising 5%–10% of all fractures and 45% of shoulder girdle injuries, predominantly affect the midshaft (approximately 80% of cases). ^1– 3^ Historically managed nonoperatively with slings or figure-of-8 braces because of reported low nonunion rates, displaced midshaft fractures now show higher nonunion (22–44%), malunion, and dissatisfaction with conservative care. ^4^ This has spurred a shift toward surgical intervention, notably plate fixation. Precontoured locking plates enhance stability, enable early mobilization, and lower nonunion risk, with added benefits of procedural ease and reduced soft tissue irritation. ^5^ Although studies worldwide have reported strong outcomes with this approach, ^6, 7^ evidence from the Middle East, particularly Yemen, remains scarce, limiting region-specific insights.

This study evaluated the functional outcomes and complications of pre-contoured superior clavicle locking plates for displaced midshaft clavicular fractures in a Yemeni cohort, focusing on patient satisfaction, range of motion, union time, and complication rates.

## Methods

### Study design

This prospective observational study was conducted at the Department of Orthopedics, Al-Thawra Modern General Hospital, and Kuwait University Hospital, Sana’a, Yemen, between January 2018 and September 2024. The primary aim of this study was to assess the functional outcomes of displaced midshaft clavicular fractures treated with open reduction and internal fixation (ORIF) using pre-contoured superior clavicle locking plates. A standardized protocol governed patient selection, surgery, postoperative care and follow-up to ensure consistency. Data were collected prospectively.

### Study population

Eligible patients were adults (≥18 years) with closed, displaced midshaft clavicular fractures, defined as displacement >2 cm, shortening >2 cm, comminution, or skin tenting threatening viability. The exclusion criteria were open or pathological fractures, proximal or distal third clavicle involvement, head or neurovascular injuries, acromioclavicular dislocations, and prior nonunion. All participants provided written informed consent.

### Sample size

The sample size was calculated based on the primary outcome, the University of California, Los Angeles (UCLA) Shoulder Score. The calculation aimed to ensure sufficient power to detect a clinically meaningful outcome. Based on a Minimal Clinically Important Difference (MCID) of approximately 4 points for the UCLA score (standard deviation 5), as suggested by prior literature on shoulder pathology, ^8, 9^ we determined that a minimum of 33 patients would be required to achieve 90% power with α = 0.05. To account for potential dropouts and to enhance the power for subgroup analyses, we aimed for a larger cohort and ultimately enrolled 65 patients.

### Surgical technique

Preoperative workup included standard blood tests and clavicular radiographs (anteroposterior, 20° cranial tilt). Under general anesthesia, the patients were positioned in a beach-chair setup with a scapular sandbag for reduction. Prophylactic antibiotics were administered before the incision. A transverse incision below the fracture exposed the clavicle via superior retraction, thereby avoiding wound overlap with the plate. Subcutaneous tissue and platysma were mobilized together, myofascial layers were incised, and soft tissues were elevated, preserving supraclavicular nerves unless exposure demanded sacrifice. Fracture reduction was performed using clamps or indirect techniques, as confirmed by fluoroscopy. A 3.5-mm titanium precontoured locking plate (Orthomed E, Egypt) was fixed anterosuperiorly with locking screws, ensuring a minimum of **three bicortical screws (i.e., six cortices) per main fragment**; lag screws were used to address butterfly fragments as needed. The fascia was repaired over the plate, the skin was closed in layers, and the arm was slung postoperatively.

### Postoperative care and follow-up

Patients received analgesics, postoperative radiographs, and sling immobilization for 4 weeks with pendulum exercises. Follow-ups at 10 days (suture removal), 4, 8, 12, and 26 weeks included a rehabilitation protocol starting week 4 (active-assisted motion, progressing to full range by week 8). UCLA scores were assessed at 6 months by two orthopedic surgeons, with inter-rater reliability assessed. Radiographic union was monitored descriptively by the treating surgeon, with delayed union defined as a lack of three-cortex bridging at 12 weeks.

### Outcome measures

The primary outcome was functional recovery assessed using the UCLA Shoulder Score (max 35), evaluating pain (0–10), function (0–10), active forward flexion (0–5), strength (0–5), and satisfaction (0–5). ^10^ Scores ≥27 indicated good/excellent outcomes, and <27 fair/poor. Preoperative scores were not recorded; however, pre-injury shoulder function was queried to exclude prior pathology. UCLA scores were calculated using the MDCalc online tool ( MDCalc UCLA Shoulder Score Calculator). The secondary outcomes included complication rates (hardware irritation, failure, infection, delayed union, and malunion).

**Bias control and variability management**

To minimize bias, UCLA Shoulder Scores were independently assessed by two orthopedic surgeons who were uninvolved in patient treatment. Inter-rater reliability was evaluated using the Intraclass Correlation Coefficient (ICC). Based on established guidelines, an ICC ≥0.80 indicates **good reliability**. Discrepancies greater than 2 points were reviewed by a third evaluator, and final scores were determined by consensus. Variability control was ensured through a standardized sample size calculation, a uniform follow-up schedule (10 days, 4, 8, 12, and 26 weeks), and a consistent rehabilitation protocol. All procedures followed a predefined surgical technique using precontoured superior clavicle locking plates, minimizing technical variations.

### Statistical analysis

Data were analyzed using SPSS version 26. Descriptive statistics summarized demographic characteristics, injury details, and outcomes (mean ± SD for continuous variables; frequencies/percentages for categorical variables). Normality was assessed using the Shapiro-Wilk test. Given the non-normal UCLA score distribution, nonparametric tests (Kruskal-Wallis, Mann-Whitney U) were used to assess subgroup differences. Chi-square tests were used to examine complication associations, and Spearman’s correlation was used to evaluate age-outcome links.

## Results

### Demographic and injury characteristics

**Figure 1.**
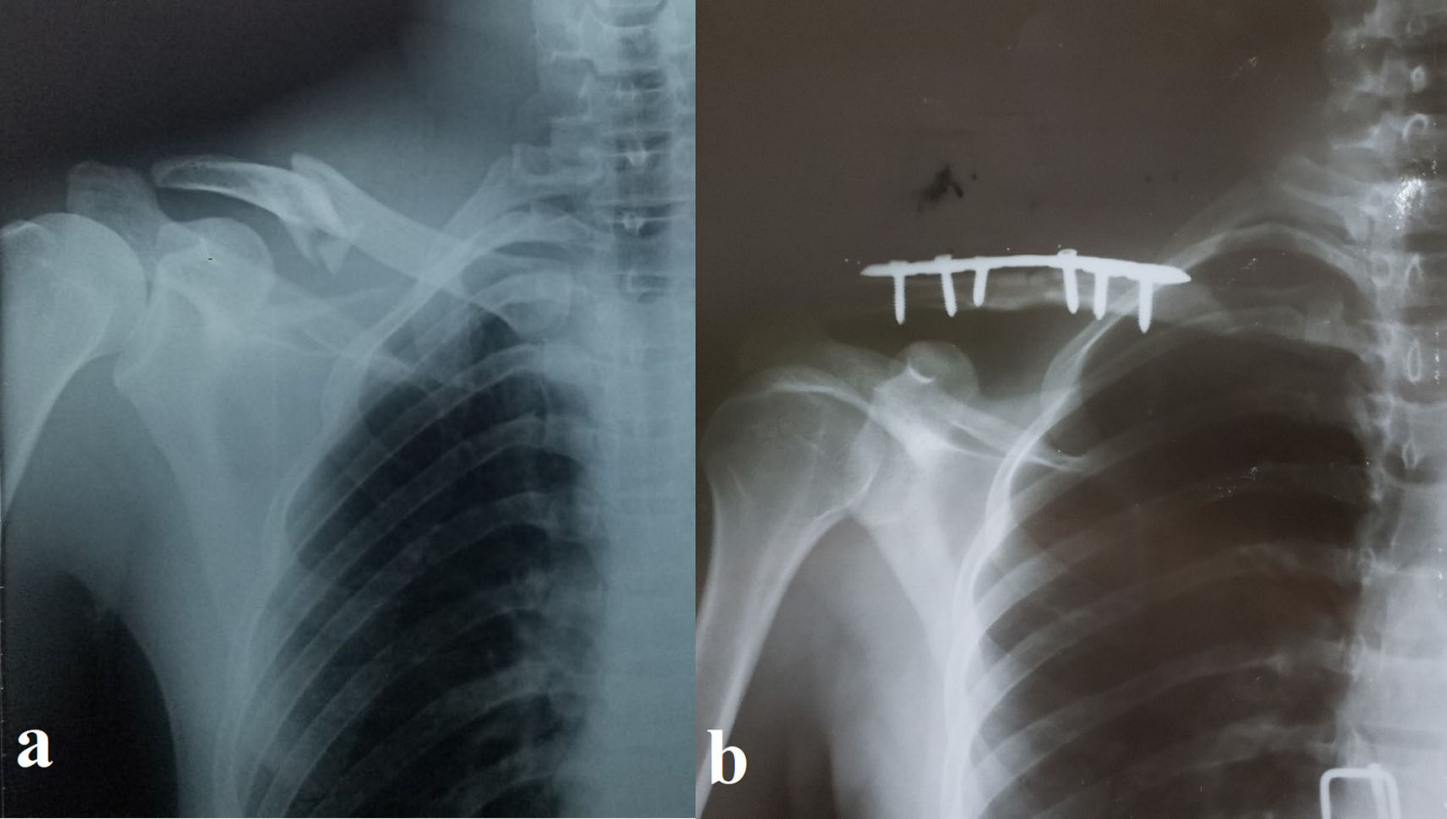
Pre- and postoperative radiographs of a 30-year-old male with displaced midshaft clavicle fracture. (a) Preoperative anteroposterior radiograph showing a displaced, slightly comminuted midshaft fracture of the right clavicle in a 30-year-old male patient. (b) Postoperative anteroposterior radiograph of the same patient following open reduction and internal fixation (ORIF) with a pre-contoured superior locking plate (Orthomed E, 7-hole, titanium). Note the anatomical reduction of the fracture and the use of locking screws that engage at least four cortices on either side of the fracture.

**Figure 2.**
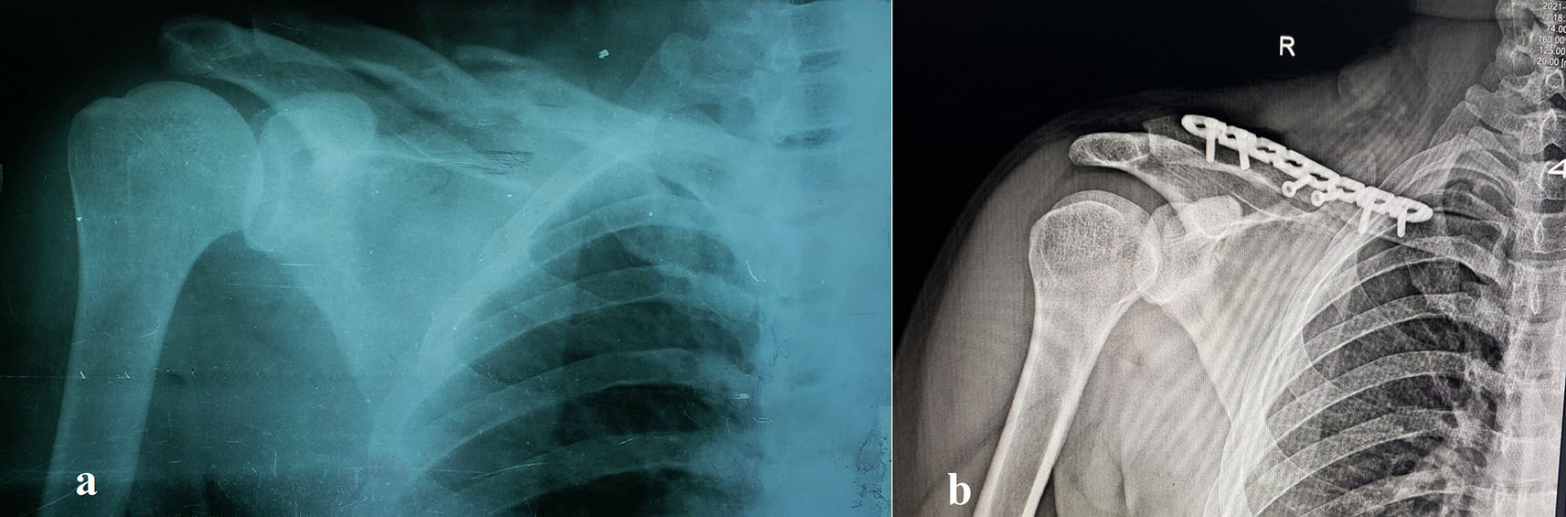
Pre- and postoperative radiographs of a 26-year-old female with comminuted midshaft clavicle fracture. (a) Preoperative anteroposterior radiograph showing a displaced, severely comminuted midshaft fracture of the right clavicle in a 26-year-old female patient. (b) Postoperative anteroposterior radiograph of the same patient following open reduction and internal fixation (ORIF) with a pre-contoured superior locking plate (Orthomed E, 8-hole, titanium). Note the anatomical reduction of the fracture and the use of locking screws that engage at least four cortices on either side of the fracture. Two lag screws were used to stabilize the butterfly fragments.

Sixty-five patients with displaced midshaft clavicular fractures treated with precontoured locked plates were enrolled. The mean age was 32.09 years (range: 19–50 years), with 54 males (83.1%) and 11 females (16.9%). Road traffic accidents (RTAs) caused the most injuries (n=43, 66.2%), followed by falls (n=22, 33.8%). The right clavicle was affected in 43 (66.2%) patients and the left clavicle in 22 (33.8%). Table 1 details these traits, and Figures 1– 2 show representative cases.

**Table 1.** Demographic and injury characteristics.

Variable	n	%
**Age group**		
18-30 years	37	56.9%
31-40 years	12	18.5%
41-50 years	16	24.6%
**Gender**		
Male	54	83.1%
Female	11	16.9%
**Mode of injury**		
RTA	43	66.2%
Accidental fall	22	33.8%
**Injured side**		
Right	43	66.2%
Left	22	33.8%

**Abbreviations:** n = number of patients; % = percentage; RTA = road traffic accident.

### Functional and radiographic outcomes

At 6 months, the mean UCLA shoulder score was 32.46 ± 2.54, with 64 patients (98.5%) achieving good/excellent outcomes (UCLA ≥27) and 1 (1.5%) fair/poor (<27). Table 2 summarizes the scores, and Supplementary Table S1 breaks down the components (Extended Data). Radiographs confirmed union in all cases by 12 weeks (three-cortex bridging), with no delayed unions or malformations noted.

**Table 2.** UCLA shoulder rating score outcomes.

Category	Value	n	%
Total UCLA Score (Mean ± SD)	32.46 ± 2.54		
**Outcome categories**			
Good/excellent (≥27)		64	98.5%
Fair/poor (<27)		1	1.5%

**Abbreviations**: UCLA, University of California, Los Angeles; SD, Standard Deviation.

### Patient satisfaction

All 65 patients (100%) reported satisfaction with surgical outcomes, reflected in the UCLA Shoulder Rating Score satisfaction component (all scored 5, “Satisfied and better”; Supplementary Table S1(Extended Data)).

### Postoperative complications

Complications were rare and included hardware irritation (n=1, 1.5%), hardware failure (n=2, 3.1%), and superficial infection (n=1, 1.5%), totaling 6.2% of patients. No delayed unions, malunions, or refractures occurred, and 61 patients (93.8%) were complication-free. Table 3 presents the outcomes.

**Table 3.** Postoperative complications.

Complication	n	%
Hardware irritation	1	1.5%
Hardware failure	2	3.1%
Superficial infection	1	1.5%
No complications	61	93.8%

### Subgroup and correlation analyses

**UCLA scores and patient characteristics**

To assess differences in total UCLA scores across subgroups, we performed Kruskal-Wallis tests for age groups (more than two categories) and Mann-Whitney U tests for sex, injury mechanism, and injured side (two categories each). Spearman’s rank-order correlation was used to examine the relationship between age (a continuous variable) and total UCLA scores. The results are summarized in Table 4.

**Table 4.** Comparison of total UCLA scores among subgroups.

Variable	Groups compared	Test statistic	p-value
Age group	18-30, 31-40, 41-50	H(2) = 16.525	< 0.001
Mechanism of injury	RTA vs. Fall	U = 319.00	0.029
Gender	Male vs. Female	U = 248.00	0.380
Injured side	Right vs. Left	U = 203.50	< 0.001

**Note:** Kruskal-Wallis tests were used for age group comparisons; Mann–Whitney U tests were used for comparisons between two groups.

**Abbreviations**: H, Kruskal-Wallis test statistic; U, Mann-Whitney U test statistic; RTA, Road Traffic Accident.

A statistically significant negative correlation was found between age and the total UCLA score (ρ = -0.317, p = 0.010), indicating that older patients tended to have lower UCLA scores. Significant differences in the total UCLA scores were also found based on the mechanism of injury (p = 0.029) and the injured side (p < 0.001). Patients with RTA injuries had higher UCLA scores than those with falls, and those with right-sided injuries had higher UCLA scores than those with left-sided injuries. No significant differences were observed according to sex.

**UCLA component scores and age**

Kruskal-Wallis tests revealed significant differences across age groups for the pain score (p=0.001) and active forward flexion (p=0.013). Post-hoc tests indicated that the 41-50 age group had significantly better scores (less pain and better flexion) than the 18-30 age group. No significant differences were observed between the age groups in terms of the function, strength, or satisfaction components of the UCLA score.

**Complications and patient characteristics**

Chi-square tests of independence were performed to examine the relationship between categorical variables (age group, mechanism of injury, sex, injured side, and UCLA outcome category) and the occurrence of complications. The results are summarized in Table 5. A statistically significant association was found between UCLA outcome category and complications (p < 0.001), with patients experiencing complications having worse outcomes, as expected. The Mann–Whitney U test revealed a statistically significant difference in the total UCLA scores between patients with and without complications (U = 11.000, p < 0.001). Patients with complications had significantly lower UCLA scores than those without complications. Although not statistically significant at p < 0.05, there were trends suggesting possible associations between age group (p=0.051, with a significant linear association p=0.046), mechanism of injury (p=0.073), and injured side (p=0.073) with complication rates.

**Table 5.** Association between categorical variables and complications.

Variable	Complications		Total	Chi-Square/U	p-value
	No, n (%)	Yes, n(%)			
**Age group**				χ ^2^(2) = 5.946	0.051
18-30 years	36 (97.3%)	1 (2.7%)	37		
31-40 years	12 (100%)	0 (0.0%)	12		
41-50 years	13 (81.3%)	3 (18.8%)	16		
Linear by linear	-	-	-	χ ^2^(1) = 3.999	0.046
**Mechanism of injury**				χ ^2^(1) = 3.224	0.073
RTA	42 (97.7%)	1 (2.3%)	43		
Fall	19 (86.4%)	3 (13.6%)	22		
**Gender**				χ ^2^(1) = 3.317	0.069
Male	52 (96.3%)	2 (3.7%)	54		
Female	9 (81.8%)	2 (18.2%)	11		
**Injured side**				χ ^2^(1) = 3.224	0.073
Right	42 (97.7%)	1 (2.3%)	43		
Left	19 (86.4%)	3 (13.6%)	22		
**UCLA outcome**				χ ^2^(1) = 15.488	< 0.001
Good/excellent (≥27)	61 (95.3%)	3 (4.7%)	64		
Fair/poor (<27)	0 (0.0%)	1 (100%)	1		
**Total UCLA**	-	-	-	U = 11.00	< 0.001

**Note:** Chi-square tests of independence were used to examine the association between categorical variables and the presence/absence of complications, reporting Pearson’s chi-square values unless otherwise noted. The Mann–Whitney U test was used to examine significant differences UCLA scores and complications.

**Abbreviations**: UCLA, University of California, Los Angeles; RTA, Road Traffic Accident; U, Mann-Whitney U test statistics.

## Discussion

This prospective study evaluated precontoured locked plate fixation for displaced midshaft clavicular fractures in a Yemeni population and observed promising functional outcomes, high patient satisfaction, and a low complication rate. The mean UCLA shoulder rating score of 32.46 at 6 months (98.5% good/excellent results) is consistent with previous reports on plate fixation efficacy. Ethiraj et al. (2016) and Itagi and Kalaskar (2020) documented similarly strong Constant-Murley scores (85.23–97.8). ^11, 12^ Moreover, our results align with high-level evidence from the **Canadian Orthopaedic Trauma Society (2007)**, whose multicenter randomized trial established that surgical fixation results in significantly improved functional outcomes and a lower rate of nonunion versus nonoperative treatment. ^13^ Our study therefore adds robust, region-specific evidence to the literature supporting surgical intervention for displaced fractures.

Subgroup analyses revealed notable patterns that merit further exploration. A significant negative correlation between age and UCLA scores (ρ = -0.317, p = 0.010) suggested that older patients (41–50 years) achieved slightly lower overall function but paradoxically outperformed younger patients (18–30 years) in pain relief and forward flexion (p = 0.001, p = 0.013). Chue et al. (2018) noted comparable trends, with better pain outcomes in older adults being attributable to lower baseline demands or greater relative gains post-ORIF. ^14^ In our cohort, younger patients, often active males who were injured in RTAs, may face higher recovery expectations, thereby driving subtle dissatisfaction despite healing. Alternatively, age-related differences in soft tissue resilience or rehabilitation adherence could play a role although pre-injury function was not quantified in this study. These contradictions highlight the need for tailored outcome metrics across age groups.

Equally compelling differences in injury mechanisms and laterality were observed. Patients with RTA injuries outperformed those with falls (p = 0.029), and right-sided injuries surpassed left-sided injuries (p < 0.001). Sharma et al. (2021) linked high-energy trauma (e.g., RTAs) to better ORIF outcomes, possibly due to stricter postoperative care, ^15^ but falls in our study varied in severity, muddying this explanation. Instead, RTA fractures might involve distinct comminution patterns, stabilized effectively by locked plates, whereas falls could skew toward simpler breaks with unrecognized soft tissue impacts. Laterality findings are equally provocative; if most patients are right-handed (data unavailable), dominant-side injuries might spur greater rehabilitation effort, yielding higher symmetry indices, as seen in Riemann et al. (2023). ^16^ Left-sided repairs, potentially on non-dominant arms, might result in less patient-driven recovery focus or slight surgical adjustments (e.g., plate contouring challenges on the left clavicle curve). These hypotheses, while speculative, align with the reported variability in shoulder recovery dynamics ^7, 17, 18^ and elevate our findings beyond mere observation, warranting targeted biomechanical and behavioral studies.

Complications, although rare (6.2%), correlated strongly with poorer UCLA outcomes (p < 0.001), which is an expected finding consistent with hardware-related setbacks. Compared with the literature rates (e.g., 5–15% for irritation or failure), ^5, 6, 8, 11, 19, 20^ our results suggest that technical proficiency and patient selection minimized risks, reinforcing ORIF’s safety profile in this setting.

### Limitations

This study has several limitations that limit its conclusions. Foremost, the absence of a control group—whether nonoperative management or alternative fixation (e.g., intramedullary nailing)—precludes definitive claims about the superiority of precontoured locked plate fixation despite its promising outcomes. The 6-month follow-up period captures early recovery but misses critical long-term outcomes, such as hardware removal rates, refracture, and chronic dysfunction, which undermine durability assessments. Geographic confinement to two Sana’a, Yemeni centers restricts generalizability, as resource availability and patient profiles may differ elsewhere. Pre-injury UCLA scores were not recorded, which hindered the direct measurement of functional gains. Additionally, the 100% satisfaction rate suggests potential reporting bias or that an insufficiently granular metric—an alternative tool (e.g., DASH) might reveal variability. These constraints underscore the preliminary nature of our findings and warrant caution. Additionally, the reported 100% patient satisfaction rate, measured via the single-item component of the UCLA score, should be interpreted with caution. This finding is likely a reflection of the tool’s limited granularity rather than a perfect outcome for every patient. Even those with minor, resolvable complications may have reported overall satisfaction relative to their pre-operative condition. This suggests a potential reporting bias, and future studies should employ more comprehensive patient-reported outcome measures, such as the DASH (Disabilities of the Arm, Shoulder and Hand) score, to capture a more nuanced view of patient satisfaction. Third, while our overall sample size was robust, the subgroup analyses (e.g., by age group) were based on smaller numbers, which limits the statistical power to detect true differences and increases the risk of a Type II error. The findings from these subgroup comparisons should therefore be considered exploratory.

Larger multicenter studies with longer follow-up periods and control groups are needed to confirm these results and assess long-term efficacy. Exploring subgroup differences (e.g., age, mechanism, and laterality) using biomechanical and patient-reported data could refine treatment strategies.

## Conclusion

This prospective study of 65 Yemeni patients with displaced midshaft clavicular fractures treated via ORIF with pre-contoured locked plates revealed promising early outcomes: a mean UCLA score of 32.46 at 6 months (98.5% good/excellent), radiographic union by 12 weeks, universal patient satisfaction (100%), and a 6.2% complication rate. These findings suggest that this approach is feasible in our cohort, particularly when early mobilization is prioritized. However, the observational design—lacking a control group (e.g., non-operative or nailing)—and 6-month follow-up constrain claims of superiority or long-term benefit, compounded by an unbalanced satisfaction metric. Larger controlled trials with extended follow-up periods are crucial to confirm these results, assess the durability, and guide the management of such fractures.

### Ethical approval and consent

This study was conducted in accordance with the ethical standards of the Institutional Review Board (IRB) of Al-Thawra Modern General Hospital, Sana’a, Yemen, and adhered to the principles of the Declaration of Helsinki (1964) and its later amendments. Ethical approval was obtained from the IRB of Al-Thawra Modern General Hospital before the commencement of the study (Reference Number: IRB-TMGH-2017-047; Approval Date: 15 November 2017). Written informed consent was obtained from all participants prior to their inclusion in the study. Participants were informed about the study’s purpose, procedures, potential risks, and benefits, and their consent was documented. Additionally, all participants provided written consent for the publication of their anonymized data, including radiographic images, after reviewing a summary of the study contents.

### Patients consent

All participants provided written consent for publication of their anonymized data, including images, after reviewing a summary of the study contents.

## Data Availability

**Figshare** **Title**: *Functional Outcomes of Displaced Midshaft Clavicular Fractures Treated with Precontoured Locked Plates: Extended Data* **DOI**: https://doi.org/10.6084/m9.figshare.28559558.v3. ^21^ This dataset includes anonymized clinical and functional outcome data, postoperative complications, and UCLA scores of patients treated during this study. The following extended data files are included in this repository:
•
**Data_Dictionary.docx** – Explanation of variables and coding schema.dataset.csv Data are available under the terms of the Creative Commons Attribution 4.0 International license (CC-BY 4.0). **Figshare** **Title**: *Functional Outcomes of Displaced Midshaft Clavicular Fractures Treated with Precontoured Locked Plates: Extended Data* **DOI**: https://doi.org/10.6084/m9.figshare.28559558.v3. ^21^ **License:** Creative Commons Attribution 4.0 International (CC-BY 4.0), allowing unrestricted reuse with proper attribution
•
**Supplementary Table S1. docx–UCLA** (Refer extended data) Shoulder Rating Score Component Breakdown. Data are available under the terms of the Creative Commons Attribution 4.0 International license (CC-BY 4.0). **Figshare** **Title**: *Functional Outcomes of Displaced Midshaft Clavicular Fractures Treated with Precontoured Locked Plates: Extended Data* **DOI**: https://doi.org/10.6084/m9.figshare.28559558.v3. ^21^ This study follows the **STROBE (Strengthening the Reporting of Observational Studies in Epidemiology) guidelines** for reporting observational research. The **completed STROBE checklist** is available in **Figshare** under the title: *“STROBE Checklist for ‘Functional Outcomes of Displaced Midshaft Clavicular Fractures Treated with Precontoured Locked Plates: A Prospective Study’”.* Data are available under the terms of the Creative Commons Attribution 4.0 International license (CC-BY 4.0).
