# Supplementary material for: Functional Outcomes of Displaced Midshaft Clavicular Fractures Treated with Precontoured Locked Plates: A Prospective Study
Source: F1000Res. 2025 Aug 28;14:374. Originally published 2025 Apr 1. [Version 3] doi: 10.12688/f1000research.162891.3 (PMC12405840; doi:10.12688/f1000research.162891.3)
Supplement: Peer review history

# Reviewer report: Reviewer response for version 3

**DOI:** 10.5256/f1000research.186918.r409699

**Published:** 2025-09-02

**Authors:** Elisa Belluzzi

**Affiliations:** 1 University-Hospital of Padova, Padova, Italy

The manuscript improved and can be accepted.

Is the work clearly and accurately presented and does it cite the current literature?

Partly

If applicable, is the statistical analysis and its interpretation appropriate?

Partly

Are all the source data underlying the results available to ensure full reproducibility?

Yes

Is the study design appropriate and is the work technically sound?

Yes

Are the conclusions drawn adequately supported by the results?

Yes

Are sufficient details of methods and analysis provided to allow replication by others?

No

Reviewer Expertise:

Clinical research, Musculoskeleta diseases

I confirm that I have read this submission and believe that I have an appropriate level of expertise to confirm that it is of an acceptable scientific standard.

# Reviewer report: Reviewer response for version 2

**DOI:** 10.5256/f1000research.183966.r401042

**Published:** 2025-08-19

**Authors:** Elisa Belluzzi

**Affiliations:** 1 University-Hospital of Padova, Padova, Italy

My comments are as follows:

 Introduction is very short. It should be improved. Please update also references.

 Authors reported that “Historically managed nonoperatively with slings or figure-of-8 braces

 because of reported low nonunion rates, displaced midshaft fractures now show higher nonunion (22–44%), malunion,

 and dissatisfaction with conservative care.” However, there are published studies reporting a lower nonunion rate (for example: Tagliapietra J, et al., 2020 [Ref 1]).

 Methods

 Please explain the design of the study. Is this a prospective cohort study?

 Please follow the STROBE guidelines for observational studies.

 Please move the aim of the study at the end of the introduction.

 Authors reported “A standardized protocol governed patient selection, surgery, postoperative care and follow-up to ensure consistency.” This sentence is unclear. Authors should report the protocol used in detail.

 The sentence “Data were collected prospectively” should be deleted. Since the authors have already stated that this is a prospective study, it is redundant to mention that data were prospectively collected.

 Why did authors select to include patients with displacement >2 cm, shortening >2 cm, comminution, or skin tenting threatening viability? Please explain.

 It is unclear why authors reported UCLA score in postoperative care and follow-up section.

 In the *Outcome Measures* section, the authors report the use of the UCLA score and cite a 1981 reference. Could the authors clarify whether they used the original English version of the score or a validated translation? This information is important for assessing the appropriateness and reliability of the outcome measure in their study population.

 It seems that authors evaluated only the UCLA score and radiographic union as outcomes. Is that correct? Please clarify all the outcomes evaluated in the outcome section, including follow-up times.

 Authors reported “Variability control was ensured through a standardized sample size calculation, a uniform follow-up schedule (10 days, 4, 8, 12, and 26 weeks), and a consistent rehabilitation protocol.” Again, this sentence is unclear. This part is present also in Postoperative care and follow-up section. Please avoid repeating the same information. Please clarify the rehabilitation protocol used.

 SPSS version 26 should be cited as suggested by the producer of the software.

 Please add a flowchart of patients selection at the beginning of the results section.

 Please add BMI in table 1.

 Figures should be better prepared. Each panel should have the same dimensions.

 In the results section authors reported also correlations. However, correlations are lacking in the statistical analysis section.

 While chi-square tests provide useful univariate associations, a multivariate analysis—such as logistic regression—would be more appropriate to identify independent predictors of complications, especially considering the potential confounding among variables like age, sex, and mechanism of injury. The same for the analysis of UCLA score. Please add.

 In the discussion authors reported the they found “high patient satisfaction”. However, satisfaction seems to be lacking in the results.

 Discussion should be improved. The results obtained should be better discussed with published papers. For example, authors cited only one study for age and UCLA scores. Are there other studies? Please add.

 It is unclear why Canadian Orthopaedic Trauma Society (2007) is written in bold.

Is the work clearly and accurately presented and does it cite the current literature?

Partly

If applicable, is the statistical analysis and its interpretation appropriate?

Partly

Are all the source data underlying the results available to ensure full reproducibility?

Yes

Is the study design appropriate and is the work technically sound?

Yes

Are the conclusions drawn adequately supported by the results?

Yes

Are sufficient details of methods and analysis provided to allow replication by others?

No

Reviewer Expertise:

Clinical research, Musculoskeleta diseases

I confirm that I have read this submission and believe that I have an appropriate level of expertise to confirm that it is of an acceptable scientific standard, however I have significant reservations, as outlined above.

## References

[1] : Midshaft Clavicle Fractures Treated Nonoperatively Using Figure-of-Eight Bandage: Are Fracture Type, Shortening, and Displacement Radiographic Predictors of Failure?. *Diagnostics* .2020;10(10) : 10.3390/diagnostics10100788 10.3390/diagnostics10100788 PMC759959733027989

# Reviewer report: Reviewer response for version 2

**DOI:** 10.5256/f1000research.183966.r394763

**Published:** 2025-08-11

**Authors:** Jing-Xin Zhao

**Affiliations:** 1 Chinese PLA General Hospital, Beijing, China

Thanks very much for the author's revisions. I have no further comments on the revised version. Congratulations to the authors!

Is the work clearly and accurately presented and does it cite the current literature?

Yes

If applicable, is the statistical analysis and its interpretation appropriate?

Partly

Are all the source data underlying the results available to ensure full reproducibility?

No

Is the study design appropriate and is the work technically sound?

Partly

Are the conclusions drawn adequately supported by the results?

Partly

Are sufficient details of methods and analysis provided to allow replication by others?

Yes

Reviewer Expertise:

Orthopedics

I confirm that I have read this submission and believe that I have an appropriate level of expertise to confirm that it is of an acceptable scientific standard.

# Reviewer report: Reviewer response for version 1

**DOI:** 10.5256/f1000research.179162.r384316

**Published:** 2025-06-16

**Authors:** Jing-Xin Zhao

**Affiliations:** 1 Chinese PLA General Hospital, Beijing, China

This article reports the treatment experience of clavicle fracture fixation with plates in the Yemen region. After reading this article, I have the following suggestions that can be used to improve it.

 1. There is no control group. What is the basis for calculating the sample size? Assuming a 4-point difference (standard deviation 5), α = 0.05, and 90% power, at least 33 patients are required. Who is the difference between?

 2. "Locking screws (≥ 3 cortical layers per fragment)"

 What is the basis for fixing each bone fragment with 3 layers of cortical bone?  Our common sense suggests that at least 4 layers of cortical bone should be used for fixation.

 3. "The inter-rater reliability was evaluated using the Intraclass Correlation Coefficient (ICC), with an ICC of ≥ 0.80 indicating excellent agreement."

 What is the basis for "ICC ≥ 0.80 indicates excellent agreement"? The literature mentions "substantial" rather than "excellent". There is a difference between the two.

 4. Refer to Table 3.

 All 65 patients (100%) reported satisfaction with the surgical outcomes. Why would there still be 100% satisfaction when there were complications?

 5. Refer to "UCLA component scores and age"

 No significant differences were observed in terms of function, strength, or satisfaction. Between what?

 Furthermore, subgroup analysis was performed with Age group: 18-30, 31-40, 41-50. What is the basis for this? Is the sample size sufficient?

 6. [Discussion]

 reported UCLA means above reinforcing our findings. This literature is about intramedullary nails, not steel plates.

Is the work clearly and accurately presented and does it cite the current literature?

Yes

If applicable, is the statistical analysis and its interpretation appropriate?

Partly

Are all the source data underlying the results available to ensure full reproducibility?

No

Is the study design appropriate and is the work technically sound?

Partly

Are the conclusions drawn adequately supported by the results?

Partly

Are sufficient details of methods and analysis provided to allow replication by others?

Yes

Reviewer Expertise:

Orthopedics

I confirm that I have read this submission and believe that I have an appropriate level of expertise to confirm that it is of an acceptable scientific standard, however I have significant reservations, as outlined above.
